# Mesenteric venous thrombosis as a rare complication of decompression sickness

**DOI:** 10.1186/s40792-020-0780-9

**Published:** 2020-01-16

**Authors:** Satoshi Toyota, Shigeyuki Nagata, Shinichiro Yoshino, Sota Kono, Syogo Kawanami, Syohei Maeda, Erina Kuramitsu, Michihiro Ichimannda, Satoko Nagamatsu, Seiichiro Kai, Yasuro Fukuyama, Hiroyuki Orita, Daisuke Korenaga

**Affiliations:** 1 Department of Surgery, Nakatsu Municipal Hospital, 173 Shimoikenaga, Nakatsu, Oita 871-8511 Japan; 2 Department of Surgery and Science, Graduate School of Medical Sciences, Kyushu University, Fukuoka, Japan

**Keywords:** Decompression sickness, Mesenteric venous thrombosis, Hyperbaric oxygen therapy

## Abstract

**Background:**

Decompression sickness (DCS) induced by extravascular and intravascular gas bubbles during decompression can present with varying manifestations, such as joint pain, numbness, cutaneous symptoms, and cardiopulmonary dysfunction. However, mesenteric venous thrombosis (MVT) is a rare complication of DCS. To the best of our knowledge, only a few cases have been reported, and surgical cases of MVT secondary to DCS have not yet been reported.

**Case presentation:**

A 59-year-old man who was a fisherman and recreational diver dived to a depth of 100 feet. After diving, he noted abdominal and postcervical pain and visited a community hospital. Computed tomography (CT) revealed a large amount of intravenous gas, so he was diagnosed with DCS. He was then transferred to a previous hospital, where hyperbaric oxygen therapy (HBOT) was performed. HBOT reduced the amount of venous gas, but his abdominal pain worsened, so he was transferred to our hospital. CT showed pneumatosis cystoides intestinalis. Because of the possibility of intestinal necrosis, a laparoscopic examination was performed, which revealed necrosis of the transverse colon. We therefore performed a transverse colon resection. He was discharged 36 days after the surgery and followed an uneventful postoperative course.

**Conclusions:**

DCS is likely to cause MVT. If intestinal necrosis is suspected, a laparoscopic examination may be useful for determining the diagnosis and treatment. MVT should be included as a differential diagnosis of abdominal pain that persists after HBOT.

## Background

Decompression sickness (DCS) is caused by extravascular and/or intravascular gas bubbles that develop during decompression [1] and shows a large range of manifestations [2]. The most frequent manifestation is pain, especially joint pain and muscular pain, followed by numbness and paraesthesia. In severe cases, central nervous system and cardiopulmonary dysfunction may occur. Gastrointestinal symptoms account for approximately 2.8% [1] of cases of DCS, and mesenteric venous thrombosis (MVT) is a rare complication secondary to DCS. To the best of our knowledge, only one case of MVT caused by DCS has ever been reported in 1984 [3].

We herein report a case of mesenteric venous thrombosis that occurred after diving and discuss the most appropriate treatment strategy.

## Case presentation

A 59-year-old Japanese man was transferred to our hospital because of aggravated abdominal pain after initial treatment for DCS at the previous hospital.

The patient was a fisherman and recreational diver and previously had DCS twice, which was treated conservatively both times. In addition, he was taking aspirin 100 mg/day because he had a history of percutaneous cardiac intervention for angina pectoris. He dove to a depth of 100 feet 3 days in a row using a self-contained underwater breathing apparatus (SCUBA) to search for a lost item. After surfacing, he experienced sudden abdominal and postcervical pain, so he visited a community hospital.

**Fig. 1:**
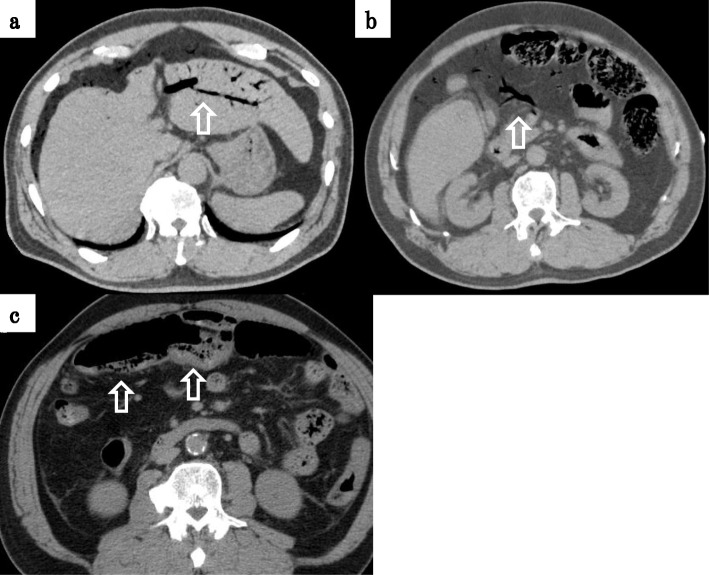
CT imaging before HBOT. **a** A large amount of intravenous gas was detected in the portal vein (white arrow) and **b** superior mesenteric vein (white arrow). **c** CT imaging after transfer to our hospital revealed pneumatosis intestinalis of the transverse colon (white arrow)

Computed tomography (CT) revealed a large amount of intravenous gas throughout his whole body, including in the portal vein (PV) (Fig. 1a), superior mesenteric vein (SMV) (Fig. 1b), inferior mesenteric vein (IMV), and femoral vein (FV). He was therefore diagnosed with DCS and transferred to the previous hospital to undergo hyperbaric oxygen therapy (HBOT). On admission to that hospital, US Navy Treatment Table 6, the most common type of HBOT, was performed. The following day, the intravenous gas had been mitigated according to the CT findings; however, pneumatosis intestinalis of the transverse colon developed. His abdominal pain remained, and he complained that the severity of the pain was worsening. Due to concerns about mesenteric ischemia, he was transferred to our hospital for additional treatment.

On arrival, he was oriented, and his vital signs were as follows: blood pressure, 123/69 mmHg; pulse rate, 120 bpm; and oxygen saturation, 93% with 3 L/min O_2_ administration. Mottling and cutis marmorata were noted on his stomach. A physical examination revealed a distended and mildly hardened abdomen, strong abdominal pain, tense muscles, and tenderness, suggesting peritonitis on palpation. The laboratory data revealed an elevated white blood cell (WBC) count (22400/μL; normal range, 4000–8500/μL) and C-reactive protein (CRP) level, hemoconcentration, acute kidney injury, acute hepatic injury, and coagulopathy (hemoglobin [Hb] 21.4 g/dL, normal range 13.0–17.0 g/dL; hematocrit [Ht] 59.9%, normal range 40.050.0%; platelet count 134000/μL, normal range 150000–300000/μL; creatinine [Cr] 3.72 mg/dL, normal range 0.61–1.04 mg/dL; aspartate aminotransferase [AST] 119 U/L, normal range 10–40 U/L; alanine aminotransferase [ALT] 127 U/L, normal range 5–40 U/L; creatine kinase [CK] 2018 U/L, normal range 58–249 U/L; CRP 17.21 mg/dL, normal range ≤ 0.30; prothrombin time [PT] 52.1%, normal range ≥ 75%; activated partial prothrombin time [APTT] 36.6 s, normal range 25–38 s; D-dimer 7.3 μg/mL, normal range ≤ 1.0 μg/mL; and fibrin degradation production [FDP] 11.8 μg/mL, normal range ≤ 5.0 μg/mL).

**Fig. 2:**
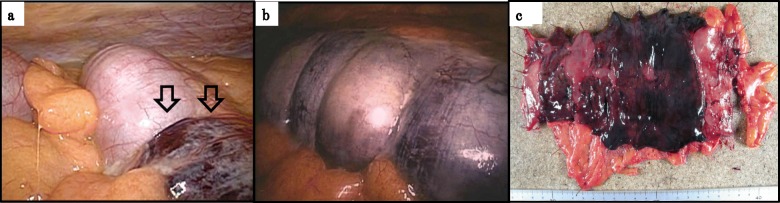
**a**, **b** Operative findings. The transverse colon and mesentery were dark red in color, suggesting mesenteric ischemia. **c** Resected transverse colon. Ischemic changes were observed in all layers

Because of his kidney dysfunction, contrast-enhanced CT was avoided, and plain CT was conducted. CT revealed pneumatosis intestinalis of the transverse colon (Fig. 1c), suggesting potential mesenteric ischemia, so we decided to perform an exploratory laparoscopy to obtain an accurate diagnosis and provide subsequent treatment. Laparoscopy revealed that the transverse colon and mesentery were dark red in color, suggesting mesenteric ischemia (Fig. 2a, b). After switching to an open laparotomy, a partial transverse colon resection and colostomy with the ascending colon were performed. In addition, we inserted a feeding tube into the jejunum to provide early nutrition after surgery. Discoloration was found in all layers of the resected specimen (Fig. 2c)

After surgery, the patient was admitted to the intensive care unit. He was on mechanical ventilation, and meropenem, a broad-spectrum antibiotic, was administered for 6 days for peritonitis and bacterial translocation. Ulinastatin, a protease inhibitor, was administered for 3 days for acute circulatory failure, and sivelestat sodium hydrate, a neutrophil elastase inhibitor, was also administered for 5 days for acute lung injury. On postoperative day 3, early nutrition was started with the feeding tube. The patient was extubated 6 days after surgery. On postoperative day 36, he was discharged in good condition. Two months later, surgery for colostomy closure was performed. He is following an uneventful course.

**Fig. 3:**
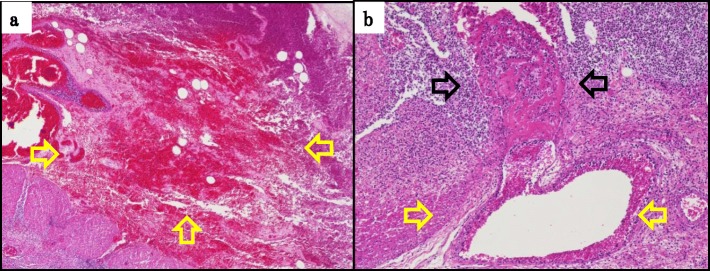
Pathological findings. **a** Diffuse edema and necrotic changes were found at the mucosal, submucosal, and muscle layers and part of the subserosa (yellow arrow). **b** In some dilated mesentery veins, there was gas embolus (yellow arrow), and thrombus formation was found near the peripheral side (black arrow)

A pathological examination revealed diffuse edema, congestion, hemorrhaging, and necrotic changes in the mucosal, submucosal, and muscle layers and part of the subserosa (Fig. 3a). In some of the dilated veins, there were a few gas embolus-like oval transparent spaces (Fig. 3b yellow arrow), and thrombus formation was found near the peripheral side (Fig. 3b black arrow). Because of these findings, he was diagnosed with MVT.

## Discussion

DCS is caused by intravascular and/or extravascular bubbles that form from dissolved inert gas during or after decompression. Decompression illness (DCI) includes DCS and arterial gas embolism (AGE) and causes pulmonary barotrauma, expands gas stretches and ruptures alveolar capillaries, allowing alveolar gas to enter the arterial circulation. DCS is distinguished from AGE by its etiology [1].

It is difficult to accurately calculate the incidence of DCS, but it is estimated that approximately 0.03% (per dive) of recreational divers are affected, according to The Divers Alert Network [1]. The mechanism underlying DCS associated with diving is as follows: while diving to depth, the partial pressure of inert gasses (mainly nitrogen) increases. According to Henry’s law, nitrogen dissolves in the tissue and blood. During ascent or after surfacing, the ambient pressure decreases (decompression), and supersaturated nitrogen may form bubbles in the tissue and blood, which can have mechanical, embolic, and biochemical effects [2].

The manifestations of DCS range from trivial to fatal, and the most common manifestation is pain, especially joint pain and muscle pain, followed by peripheral neurologic symptoms, such as numbness and paresthesia [2]. Approximately 13% [1] of divers complain of gastrointestinal disturbance upon ascent. Most divers present with aerophagy, while some patients, including our patient, have abdominal pain [4], although portal venous gas itself is generally not thought to cause pain [5]. The mechanism is not clear, but micro-congestive ischemia from mechanical embolisms and endothelial damage induces vascular hyperpermeability and hypercoagulation due to intravascular bubbles [3], which are the main cause of MVT and may be related to pain.

The diagnosis of DCS is made on a clinical basis, so an accurate history and physical examination of symptomatic individuals after diving are crucial. Imaging studies such as chest X-ray, abdominal X-ray, ultrasonography, and CT are not necessary to diagnose DCS; however, these imaging studies help determine the severity and may provide significant findings that will aid in the selection of treatment [6]. The fisherman had a history of diving for 3 days in a row with aggravated abdominal pain after diving and intravenous gasses present throughout his whole body, so he was diagnosed with DCS.

Contrast-enhanced CT is the most useful imaging tool for diagnosing MVT. The sensitivity and specificity of CT angiography are 93% and 100%, respectively [7]. The most characteristic finding of MVT is the presence of a filling defect within a mesenteric vein. Nonspecific findings include bowel wall thickening, indistinct bowel margins, ascites, and a thickened mesentery [7]. In addition, the presence of pneumatosis intestinalis and mesenteric or portal venous gas are suggestive of ischemic colitis.

The basic treatment for DCS is a rapid administration of 100% oxygen [1], which may help mitigate the symptoms, but most beneficial treatment for DCS is HBOT with 100% oxygen (recompression). A common recompression region is the US Navy Treatment Table 6 protocol, in which patients are compressed to 2.8 bar (equivalent to 18-m sea-water depth) while breathing 100% oxygen. HBOT decreases the bubble volume, as predicted by Boyle’s law, and increases the inert gas partial pressure gradient between the tissue and alveolar gas, thus ameliorating tissue hypoxia. These effects lead to the quick resolution of the bubbles, relieve mechanical pressure on the surrounding tissue, and encourage the redistribution of bubbles lodged in the microcirculation [8]. HBOT is the best treatment for DCS, and many patients achieve complete resolution with HBOT; however, some severe patients are resistant to initial treatment and require several rounds of treatment [1]. In this case, the patient underwent just one round of treatment because he developed transverse colon ischemia. The patient had aggravated abdominal pain after HBOT, the physical examination suggested peritonitis, the laboratory examination findings revealed hemoconcentration and coagulopathy, and the presence of pneumatosis intestinalis on CT dictated mesenteric ischemia. Therefore, emergent exploratory laparoscopy was performed and revealed mesenteric ischemia, and the infarcted transverse colon was resected. These surgical strategies saved the patient’s life. This first surgical report of MVT secondary to DCS is enlightening as it suggests a differential diagnosis for acute abdomen after diving.

## Conclusions

MVT can occur as a complication of DCS. In addition, in severe cases of MVT, in which peritonitis or mesenteric ischemia is suspected, exploratory laparoscopy seems to be effective for obtaining an accurate diagnosis and determining subsequent treatment. If abdominal pain persists after the initial treatment for DCS (HBOT and adequate hydration), we should consider MVT as a severe complication of DCS.

## Data Availability

Not applicable (because our manuscript is case report)

## Abbreviations

DCS: Decompression sickness
HBOT: Hyperbaric oxygen therapy
MVT: Mesenteric venous thrombosis
